# Impact of COVID-19 on University Students’ Quality of life and mental health in Greece: Apostolos Vantarakis

**DOI:** 10.1093/eurpub/ckac131.476

**Published:** 2022-10-25

**Authors:** A Vantarakis, F Fotini Tsami, A Kanellopoulou, L Alexopoulou-Prounia, A Tsapara, P Alexopoulos, K Katsadoros

**Affiliations:** 1 Public Health, Medical School, University of Patras, Patras, Greece; 2 Psychiatric Clinic, Medical School, University of Patras, Patras, Greece; 3 Klimaka, Athens, Greece

## Abstract

COVID-19 has a serious impact on people’s physical health and mental health. The COVID-19 pandemic has highlighted an increasing deterioration of university students’ quality of life and mental health due to several factors. The COVID-19 pandemic forced university students to take online classes, which may have bad impacts on students’ learning. In addition, the students lost many job opportunities during the pandemic. Faced with employment and study pressure and worried about the epidemic, university students were prone to increased overall negative emotion, anxiety and depression. Our study aims to conduct a timely assessment of the impact of the COVID-19 pandemic on the quality of life and mental health of University students. We conducted a cross sectional study using an online interview survey in students at public universities in Greece to better understand the effects of the pandemic on their quality of life and mental health. Three questionnaires were used (WHOQOL-BREF, IES-R and HADS). The data were analyzed with IBM SPSS 26. 1.266 university students from public Greek Universities participated in the study, 73.1% of which were female, 26,3% were male. We observed that 55,8% had a score lower in psychological domain and 52,3% in social domain of WHOQOL-BREF. Also 46.6% of the respondents had a score of 37+ on the IES-R questionnaire, 45% of the respondents had abnormal results regarding anxiety and 33.6% had abnormal results regarding depression. Due to the long-lasting pandemic and onerous measures such as lockdown and stay-at-home orders, the COVID-19 pandemic brings negative impacts on University education and quality of life of students. The findings of our study highlight the urgent need to develop interventions and preventive strategies to address the quality of life and mental health of University students.

**Key messages:**

• There is a need for preventative measures for university students to ensure that their mental health and quality of life do not suffer.

• Female students reached higher levels of anxiety in the COVID-19 pandemic period.

